# Immunomodulatory drug methotrexate used to treat patients with chronic inflammatory rheumatisms post-chikungunya does not impair the synovial antiviral and bone repair responses

**DOI:** 10.1371/journal.pntd.0006634

**Published:** 2018-08-03

**Authors:** Yosra Bedoui, Claude Giry, Marie-Christine Jaffar-Bandjee, Jimmy Selambarom, Pascale Guiraud, Philippe Gasque

**Affiliations:** 1 Unité mixte de recherche sur les processus infectieux en milieu insulaire tropical (PIMIT), INSERM U1187, CNRS 9192, IRD 249, Université de La Réunion—Plateforme Technologique CYROI—2, rue Maxime Rivière—Sainte-Clotilde, Île de La Réunion, France; 2 Laboratoire de biologie, secteur Laboratoire d'immunologie clinique et expérimentale de la zone de l'océan indien (LICE-OI) CHU La Réunion site Félix Guyon, Allée des Topazes, Saint Denis de La Réunion, France; 3 Laboratoire de biologie, CNR associé des arbovirus, CHU La Réunion site Félix Guyon, Allée des Topazes, Saint Denis de La Réunion, France; Fundacao Oswaldo Cruz, BRAZIL

## Abstract

Chikungunya virus (CHIKV) is a mosquito-transmitted RNA alphavirus causing major outbreaks of infectious chronic inflammatory rheumatisms (CIR). Recently, methotrexate (MTX), a disease modifying anti-rheumatic drug has been used successfully to treat patients suffering from rheumatoid-like arthritis post-CHIK but its immunomodulatory activity in the context of viral persistence has been a matter of concerns. We herein used a model of primary human synovial fibroblasts (HSF) and the synthetic molecule polyriboinosinic:polyribocytidylic acid (PIC) to mimic chronic infectious settings in the joints of CHIKV infected patients. The innate antiviral immune and inflammatory responses were investigated in response to MTX used at the therapeutic concentration of 1 μM. We found that MTX did not affect cellular viability as indicated by the LDH release assay. By quantitative RT-PCR, we observed that HSF responded robustly to PIC by increasing ISG15 and IFNβ mRNA levels. Furthermore, PIC upregulated the mRNA expression of two of the major pattern recognition receptors, RIG-I and MDA5 involved in the innate immune detection of viral RNA. MTX did not impact the antiviral response of PIC on ISG15, IFNβ, RIG-I and MDA5 mRNA expressions. MTX alone or combined with PIC did not affect the expression of proinflammatory CCL2 and CXCL8 chemokines. PIC strongly upregulated the mRNA and protein expression of osteoclastogenic factors (IL-6, GM-CSF but not RANKL). Critically, MTX treatment alone or combined with PIC did not affect the expression of all three tested osteoclastogenic cytokines. We found that MTX alone did not increase the capacity of CHIKV to infect and replicate in HSF. In conclusion, our study argues for a beneficial effect of MTX to treat CIR post-CHIKV given that it does not critically impact the antiviral, the proinflammatory and the bone tissue remodeling responses of synovial cells.

## Author summary

Chikungunya is a mosquito-borne virus (CHIKV) and has been incriminated in the development of arthralgia (pain of the joint) and arthritis particularly in elderly patients. Methotrexate (MTX) has been used widely to effectively treat these chronic rheumatic symptoms. Using a model of primary human joint fibroblasts (HSF), we investigated the capacity of the MTX immunosuppressive drug to affect the immune antiviral and inflammatory responses essential to clear the virus while allowing bone tissue repair. This study is important given that CHIKV and its RNA were shown to persist in the joint for months to years post infection and leading to injuries through ill-characterized mechanisms. The molecule PIC was used to mimic the effect of viral RNA. Interestingly, we found that MTX did not affect the expression of several proinflammatory and bone repair factors by HSF. Remarkably, MTX did not also impair the antiviral response of synovial fibroblasts. Our study revealed for the first time that MTX treatment should be considered as safe even in the context of viral persistence associated with chronic inflammation. MTX will not affect the capacity of the synovial tissue to maintain antiviral mechanism, to control inflammation and to promote bone tissue repair.

## Introduction

Alphaviruses, transmitted by bites of infected mosquitoes, are globally distributed and capable to cause significant inflammatory diseases including arthritis and encephalitis [1]. Old World alphaviruses, such as Chikungunya virus (CHIKV), Ross River virus (RRV) and O’Nyong-Nyong virus (ONNV), are associated with rheumatic diseases in humans which can be chronic and severely debilitating [2].

Classically, patients acutely infected with CHIKV present with a febrile illness, polyarthralgia, myalgia and maculopapular rash that can last for several days [3,4]. Remarkably, severe complications in adults such as persistent arthralgia and destructive arthritis have been reported, consistent with chronic inflammatory rheumatisms (CIR)[5–8]. Symptoms can persist for months or even years following initial infection.

The immunopathological mechanisms responsible for CHIK-CIR are poorly understood but may be due to viral persistence leading to chronic expression of viral RNA and with local inflammatory responses to drive osteoclastogenic activities [9–12].

Conceptually, the CHIK-CIR might be favored by different mechanisms: CHIKV can replicate in very high load and was reported to block type I interferon (IFN) regulatory pathway involved in the antiviral response [13,14]. Induction of type I Interferon (IFN α and β) by intracellular sensors also called pattern recognition receptors (PRR) such the Toll-like receptors (TLRs) and RIG-I-like receptors (RLRs), namely retinoic acid-inducible gene I (RIG-I) and melanoma differentiation-associated gene-5 (MDA5) represent an early innate immune response against viruses [15]. RIG-I and MDA5 can detect cytoplasmic dsRNA generated during viral replication and are able to bind polyriboinosinic:polyribocytidylic acid (PIC), the synthetic analog of viral dsRNA, and to mediate type I IFN responses [16]. IFN-stimulated genes (ISGs) code for antiviral proteins to inhibit virus replication [17]. ISG15 was reported to be a central player in the control of CHIKV infection [18].

The joint destructive process may also be mediated at least in part by fibroblast synoviocytes [19]. Interestingly, human synovial fibroblasts (HSFs) infected by CHIKV are able to promote differentiation of monocytes/macrophages into osteoclasts involved in bone erosion [20].The differentiation of osteoclasts is regulated in response to various cytokines, including the receptor activator of nuclear factor-Kappa B ligand (RANKL), macrophage colony stimulating factor (M-CSF), granulocyte-macrophage colony-stimulating factor (GM- CSF), interleukins (IL)-6 and IL1β [21,22]. RANKL, which is a member of tumor necrosis factor (TNF) family, has been identified as a key mediator of osteoclast activation and maturation in the presence of M-CSF [23]. It has already been reported that CHIKV patients presented high levels of RANKL and IL-6 which could participate in macrophage-derived osteoclast appearance in the joints [24,25]. During rheumatoid arthritis (RA), high levels of IL-6, IL1β and GM-CSF were described in the inflamed joints [26]. High plasma GM-CSF concentrations were also reported in patients displaying chronic symptoms after CHIKV infection [27].

Recruitment of inflammatory cellular infiltrates to the joint of infected patients has been reported during CHIKV induced arthropathy [28,29]. Chemokines such as CCL2 and CXCL8 are involved in modulating the recruitment of immune cells such as monocytes and neutrophils, in the inflamed joint and have been found to be upregulated in the serum and synovial fluid of CHIKV-infected patients [28,30]. Treatment of alphavirus-infected mice with Bindarit, an inhibitor of CCL2, CCL8 and CCL7 was able to ameliorate cellular infiltration in joints and attenuate the joint swelling, which suggest the major role of chemokines in joint damage and inflammation [24].

Methotrexate (MTX), a disease modifying anti-rheumatic drug (DMARD), has been successfully used to treat patients experiencing rheumatoid arthritis (RA)-like CIR months to years post CHIK [6,28,31,32]. Originally developed as a clinical chemotherapeutic agent for malignancies such as leukemia [33], MTX has become the first DMARD prescribed in patients with RA [34]. MTX is a potent competitive inhibitor of dihydrofolate reductase (DHFR) and is currently used once-weekly at low dose for treatment of inflammatory diseases due to its beneficial anti-inflammatory and immunosuppressive activities [33]. Immunosuppressive medications using MTX and others such as hydroxychloroquine, etanercept, adalimumab, and sulfasalazine could be detrimental in the context of viral persistence. Indeed, it has been hypothesized that these treatments may interfere with immune-mediated control of infection and the resolution of inflammation [2].

Hence, our aim has been to develop an *in vitro* model to ascertain whether or not MTX may affect the innate immune, inflammatory and osteoclastogenic responses of synovial fibroblasts in the context of CHIKV persistence in the joint of patients suffering from CIR.

To mimic cytoplasmic viral dsRNA generated in synovial tissue of chronically CHIKV infected patients, we used the synthetic analog of viral dsRNA PIC. Importantly, it has already been shown that PIC alone can drive a pro-arthritic inflammatory response in animal models of RA [35].

## Materials and methods

### Cells and reagents

The primary cultures of HSF were obtained from ScienCell Research Laboratory (ScienCell, 4700; Clinisciences). Cells were grown in Minimum Essential Medium eagle (MEM eagle, PAN Biotech P0408500) supplemented with 10% of decomplemented fetal bovine serum (FBS) (PAN Biotech, 3302 P290907) and completed with L-glutamine 2 mM (Biochrom AG, K0282), 100U/mL– 0.1 mg/mL penicillin- streptomycin (PAN Biotech, P0607100), 1 mM sodium pyruvate (PAN Biotech, P0443100) and 0.5 μg/mL fungizone (PAN Biotech, P0601001). We used primary cultures of HSF to investigate the cellular response to PIC and the immunoregulatory effect of MTX treatment. MTX was used at the concentration of 1μM which corresponds to the maximal plasma concentration after the ingestion of a 15 mg tablet of MTX recommended for the treatment of RA [36].

Lipopolysaccharide (LPS), a TLR4 agonist (cat. no. L2762) was purchased from Sigma-Aldrich. IL-1β (cat. no. 200-01B) and Tumor necrosis factor-α (TNFα) (cat. no. 300-01A) were purchased from Peprotech. LPS, and the recombinant cytokines were used as canonical proinflammatory activators of synovial fibroblasts [37]. The double-stranded polyribonucleotide PIC (cat. no.27-4732-01) was purchased from Amersham Biosciences.

We used a viral isolate (clone CHIKV 4.2) amplified from a patient’s serum sample (isolated during the 2006 epidemic) after two passages on Vero cells [28].

### Cell culture and treatment

HSF were placed in a six-well tissue culture plate and maintained at 37°C in a humid atmosphere with 5% CO2. The medium was replaced twice a week. Cells were allowed to grow to 80–90% confluence. Infections were performed with CHIKV clone 4.2 in a BSL3 facility and HSF were treated or not with MTX to evaluate MTX effects on CHIKV replication and cellular response to CHIKV infection.

To analyze the expression profile of HSF innate immune genes, proinflammatory chemokine genes and osteoclast-related cytokine genes, cells were stimulated with PIC100 μg/mL in the presence or not of MTX. After treatment, supernatants were collected and frozen at -20°C until analyzed.

The concentration of MTX used in the different experiments was 1μM and the culture periods were from 6h to 72h of continuous exposure to MTX. On the basis of pharmacokinetic analysis, the ingestion of a 15mg tablet of MTX produces plasma MTX concentrations of approximately 0.7μM (Cmax) after 1.5 hours [36]. MTX can distribute to the synovial fluid, in which the level of MTX is comparable with that in plasma [38]. MTX was also used at the concentration of 10μM to evaluate potential cytotoxic effect on HSF.

### Quantitative real-time RT-PCR (qRT-PCR)

Total RNA was extracted directly from harvested cell culture (in six well plates) using a QIAamp RNA Blood Mini Kit (QIAGEN, Cat No 52304). 350μL of lysis buffer from the kit was added to each well, collected after 5 min and kept at -80°C until use.

qRT-PCR experiments were done using the One Step Prime Script Syber Green RT-PCR kit from TAKARA (Cat No RR066A). qRT-PCR was performed in a final volume of 5μL containing 1μL of extracted total RNA per reaction, 2.7μL of enzyme mix and 1.3μL of primers mix with final primer concentration of 250nM. The specific primers used for qRT-PCR are listed in Table 1.

**Table 1:** List of primers used for qRT-PCR.

Primer name	Sequence (5'-3')	GenBank accession	Product size (bp)
Hu GAPDH_377F	GAACGGGAAGCTTGTCATCA	NM_002046.5	473
Hu GAPDH_849R	TGACCTTGCCCACAGCCTTG		
Hu RIG-I_279F	GCTATCGGGTCAACAACAGCTT	AF_092922	151
Hu RIG-I_429R	CCATATCTCAGCTGGGTGACAAA		
Hu IFNβ_573F	GTCACTGTGCCTGGACCATA	NM_002176.3	154
Hu IFNβ_726R	ACAGCATCTGCTGGTTGAAGA		
Hu ISG15_199F	AGATCACCCAGAAGATCGGC	NM_005101.3	153
Hu ISG15_351R	GAGGTTCGTCGCATTTGTCC		
Hu MDA5_1184F	CTGTTTACATTGCCAAGGATC	AF_095844	280
Hu MDA5_1463R	ACACCAGCATCTTCTCCATTT		
Hu CCL2_190F	CAATAGGAAGATCTCAGTGC	NM_002982.3	188
Hu CCL2_377R	GTGTTCAAGTCTTCGGAGTT		
Hu CXCL8_73F	CAGAGACAGCAGAGCACACA	NM_000584.3	158
Hu CXCL8_230R	GGCAAAACTGCACCTTCACA		
Hu RANKL_142F	TGATTCATGTAGGAGAATTAAACAGG	XM_017020803.1	82
Hu RANKL_223R	GATGTGCTGTGATCCAACGA		
Hu GM-CSF_332F	CTACAAGCAGCACTGCCCT	NM_000758.3	96
Hu GM-CSF_427R	AGCAGAAAGTCCTTCAGGTTC		
Hu IL-6_698F	AAAGAGGCACTGGCAGAAAA	XM_011515390.2	351
Hu IL-6_1048R	AAAGCTGCGCAGAATGAGAT		
CHIK E2_9059F	CACAACAGTCCGGCAACGTAA	KY575571.1	155
CHIK E2_9213R	TTTGTGATTGGTGACCGCG		
CHIK NSP1_264F	TGATGTCGGACAGGAAGTACCAC	KY575571.1	157
CHIK NSP1_420R	GCCATTACTGCTTGTAAGTCCCC		
Hu M CSF_443F	CCCTCCCACGACATGGCT	NM_000757.5	108
Hu M CSF_550R	CCACTCCCAATCATGTGGCT		
Hu IL1β_177F	ACAGATGAAGTGCTCCTTCCA	NM_000576.2	73
Hu IL1β_249R	GTCGGAGATTCGTAGCTGGAT		
Hu ADAM17_826F	CACCTGAAGAGCTTGTTCATCG	NM_003183.5	126
Hu ADAM17_951R	TACTCTCTTCCCCTCTGCCC		

qRT-PCR was carried out in Labgene Biometra T Optical thermocycler with the following steps: a reverse transcription at 42°C for 5 minutes and 40 cycles comprising a denaturation step at 95°C for 5 sec, annealing step at 58°C for 15sec and extension step at 72°C for 15 sec. Fluorescence data were collected at 520 nm during the extension step. Relative gene expression was calculated using GAPDH as a reference gene. Experiments were done in triplicate or in quadruplicate.

### Flow cytometry

For measurement of CD90, CD13, CD55 and CD59 surface expression, HSF were detached from 6-well plates with EDTA 5mM, washed with PBS/BSA, and incubated for one hour with the following monoclonal antibodies: phycoerythrin (PE)-conjugated anti-CD55 (1:100, BioLegend), Fluorescein isothiocyanate (FITC) conjugated anti-CD90 (1:100, BioLegend), PE anti-CD59 (1:100, BioLegend), PE anti-CD13 (1:100, BECKMAN COULTER) or isotype control antibodies: IgG1-FITC (1:100, BECKMAN COULTER) and IgG1-PE (1:100, BECKMAN COULTER). Stainings were visualized by flow cytometry with BD ACCURI flow cytometer.

### Enzyme-linked immune-sorbent assay (ELISA)

Cytokine and chemokine concentrations in supernatants of HSF were measured using commercially available ELISA kits for CCL2 (Peprotech: cat. no. 900-T31), CXCL8 (Peprotech; cat. no. 900-T18), IL-6 (Peprotech; cat. no. 900-T16), GM-CSF (Peprotech; cat. no. 900-K30), and RANKL (Peprotech; cat. no. 900-K142), according to the manufacturer’s instructions. Samples were analyzed from three to four independent experiments.

### Cytotoxicity assay

The Cytotox96 assay from Promega (cat. no.G1781) is a colorimetric-based cytotoxicity assay that quantitatively measures the release of lactate dehydrogenase (LDH) from damaged cells.CytoTox 96 Non-Radioactive Cytotoxicity Assay was used following treatment with MTX 1**μ**M and 10**μ**M. After treatments, culture medium was recovered, and then cells were lyzed following the manufacturer's instructions. Released LDH in culture medium was measured for detection of cell damage following treatments. Intracellular LDH (induced by the addition of the lysis buffer) was measured for determination of the maximum LDH release. The percentage of cellular injury was calculated using the formula: % cytotoxicity = 100 × experimental LDH release / maximum LDH release.

### Statistics

Statistical analyses were performed with GraphPad Prism software version 6.01 using a Student unpaired *t* test. p-values ≤ 0.05 were considered statistically significant. Significance was indicated in the figures as follow: p-values ≤ 0.05 (*), p-values ≤ 0.01 (**), p-values ≤ 0.001 (***) and p-values ≤ 0.0001 (****). Results are expressed as mean ± standard error “SEM” and as percentage.

## Results

### MTX used at micromolar concentration does not have cytotoxic activities on HSF

**Fig 1:**
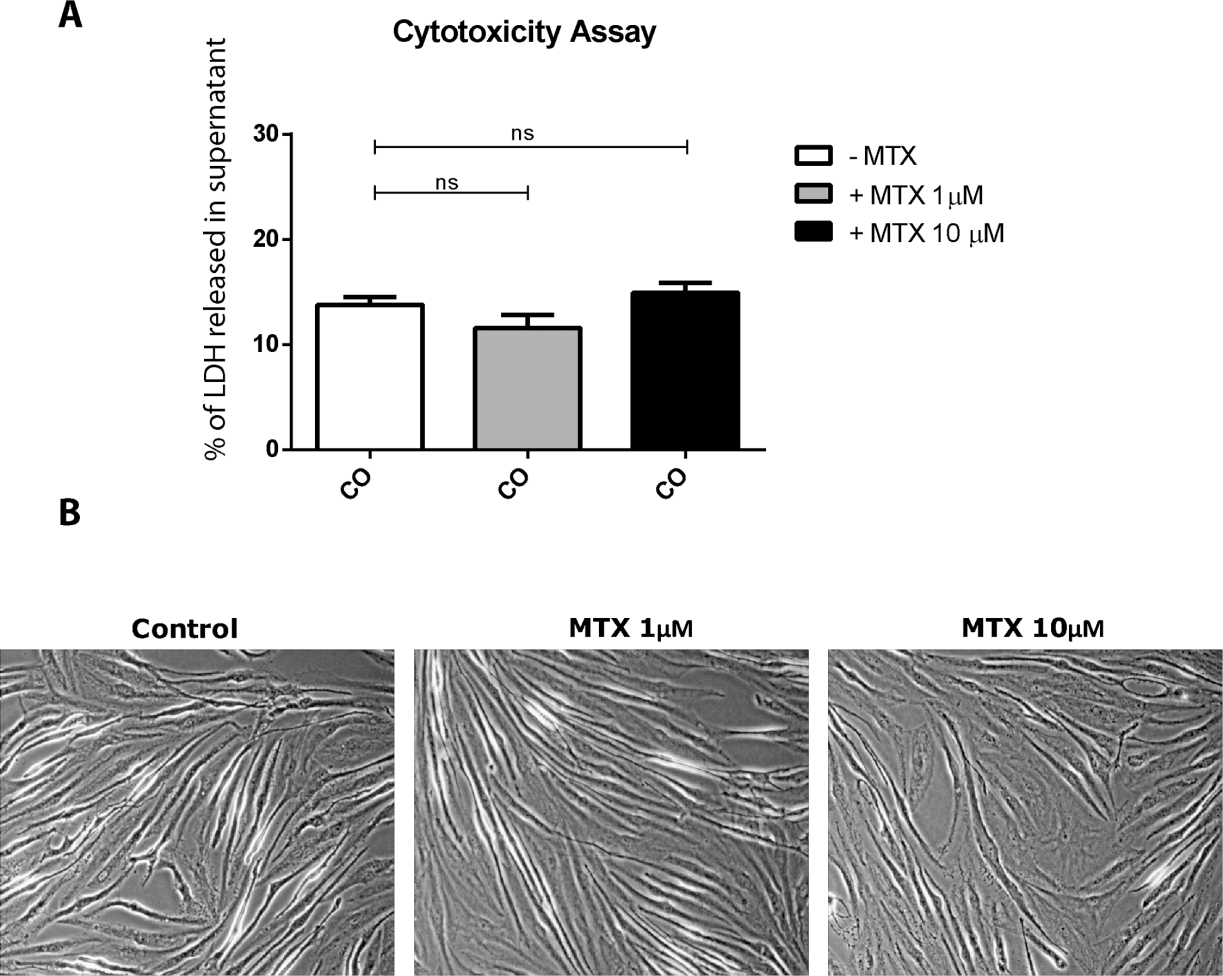
MTX at 1 μM to 10 μM does not cause HSF cytotoxicity. **A)** HSFs were treated with MTX at a concentration of 1μM or 10μM for 24 hours. Percentage of LDH released in culture supernatant was measured using the CytoTox 96 Non-Radioactive Cytotoxicity Assay. Results are from 3 independent experiments. **B)** HSF cell morphology after treatment with MTX 1μM and 10μM for 24 hours. Magnification is x100.

Control cells (medium alone) showed a basal level of cell toxicity as indicated by background levels of LDH released in HSF cell culture supernatants. MTX treatment at the concentration of 1μM and 10μM did not affect significantly the level of LDH release compared to control cells (**Fig 1**A). As shown in Fig 1B, we observed that MTX treatment did not induce cell shrinking and failed to induce necrotic activities.

### MTX treatment does not impair the antiviral innate immune response of HSF stimulated by PIC viral analog

We have evaluated by Sybergreen qRT-PCR the expression of several antiviral genes. We screened for the expression of RLRs (RIG-I, MDA5), IFN β and ISG15. We used GAPDH as a housekeeping gene and to establish the relative expression of each mRNA. Early (6h) and late (24h) regulatory mechanisms were analyzed.

**Fig 2:**
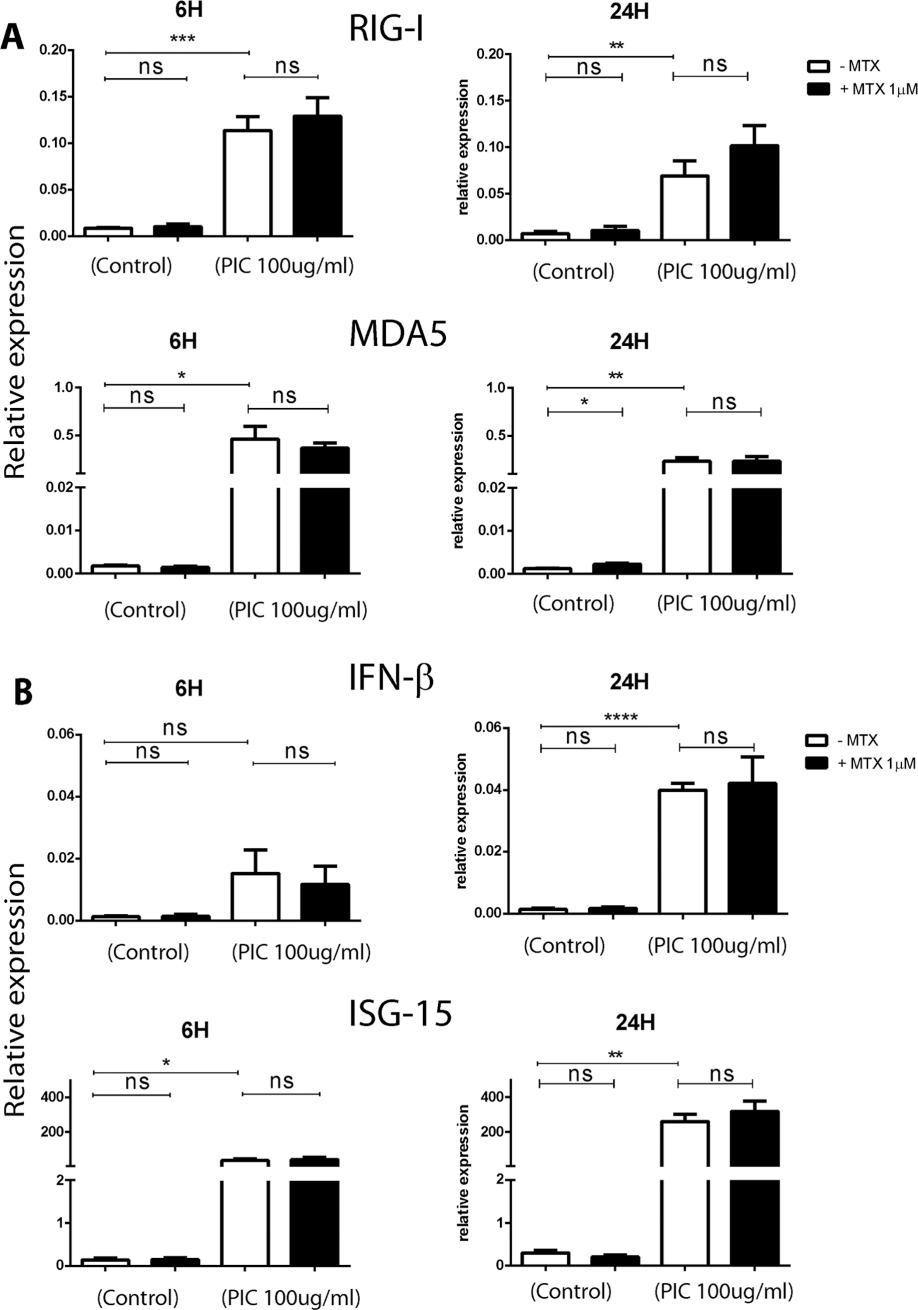
MTX treatment has no significant effect on the expression of several antiviral innate immune genes. **A)** Relative expression of PRR (RIG-I and MDA5) from HSF stimulated by PIC100μg/mL +/- MTX 1μM for 6 hours and 24 hours as assessed by qRT-PCR. **B)** IFN β and ISG15 relative expression in HSF after PIC100μg/mL +/- MTX 1μM treatment for 6 hours and 24 hours as measured by qRT-PCR. All experiments were done in quadruplicates and results are expressed as mean ± standard error. *: p-values ≤ 0.05, **: p-values ≤ 0.01, ***: p-values ≤ 0.001 and ****: p-values ≤ 0.0001.

In response to PIC stimulation (**Fig 2**A), the relative expression of RIG-I mRNA was significantly increased in HSF at 6 h (1.14x10^‐1^ ± 2.99x10^-2^, p<0.001) corresponding to a fold change of 13. MDA5 was also highly expressed in response to PIC, (4.61x 10^‐1^ ± 2.63x10^-1^, p<0.05) with a fold change of 262 when compared to control untreated cells. At 24h post PIC treatments, the levels of expression were (6.89x10^-2^ ± 3.26x10^-2^, p<0.05) for RIG-I (10 fold) and (2.3x 10^‐1^ ± 8.05x10^-2^, p<0.05) for MDA5 (197 fold) when compared to control conditions. The levels of expression in control cells were for RIG-I: 6.71x10^-3^±4.68x10^-3^; and for MDA5: 1.17x 10^‐3^ ± 2.59x10^-4^.

After MTX 1μM treatment alone, the relative expression of RIG-I and MDA5 was not significantly affected in HSF at 6h. In contrast, the expression of MDA5 was increased at 24h with a fold change of 2. More importantly, MTX did not affect the expression of RIG-I and MDA5 in response to PIC.

The level of IFN β mRNA (Fig 2B) was not significantly affected at 6h after PIC. A more significant increase up to 27 fold was observed at 24h (3.99x 10^‐2^ ± 3.75x10^-3^, p<0.0001) versus (1.48x 10^‐3^ ± 6.69x10^-4^) in control cells. The relative expression of ISG15 was significantly higher at 6h and 24h in response to PIC (more than 200 fold at 6h and 800 fold at 24 h). MTX did not affect significantly the relative expression of IFN-β and ISG15 in all tested conditions: alone or together with PIC.

### MTX treatment has no effect on the expression of proinflammatory chemokines by HSF stimulated by PIC

**Fig 3:**
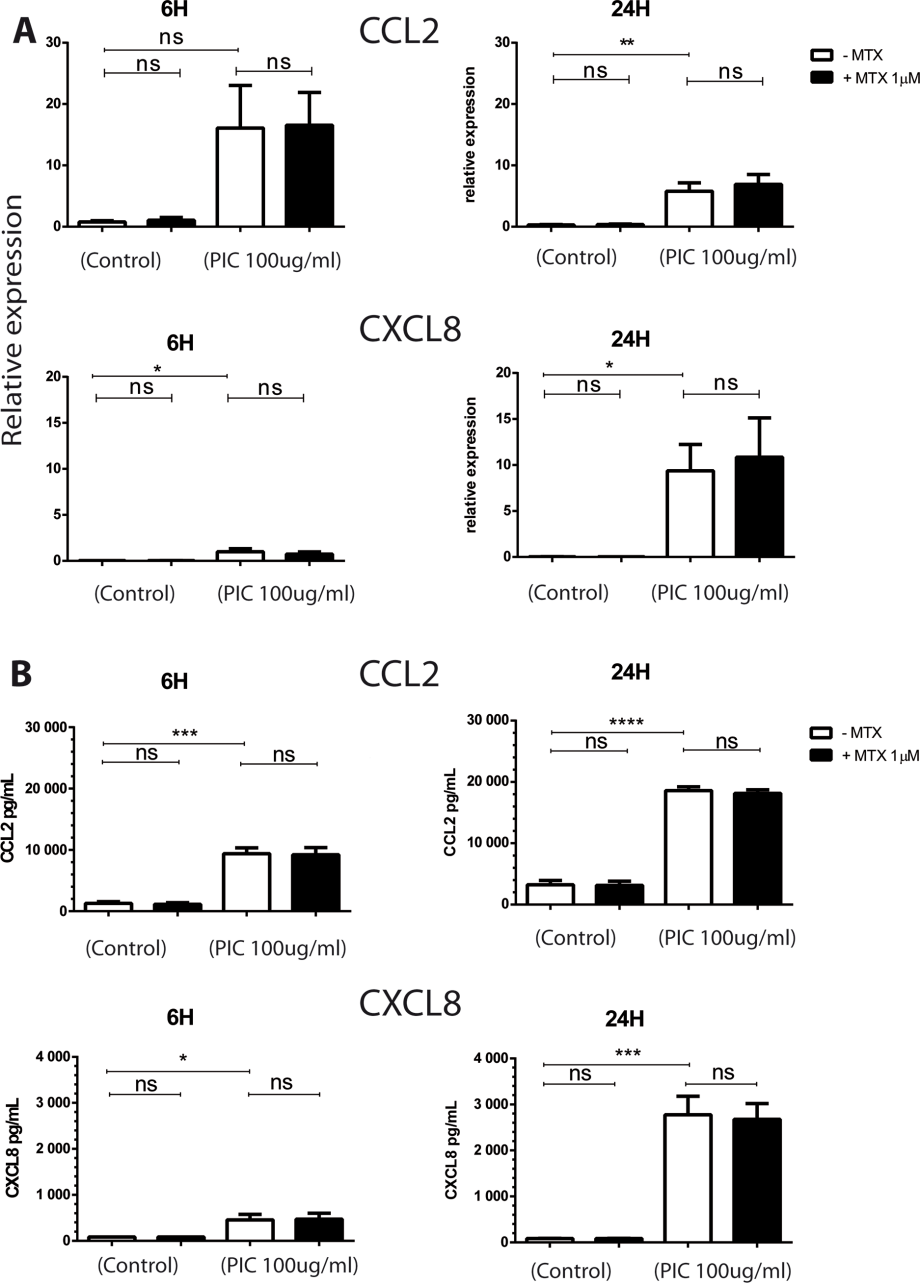
MTX effects on the expression of proinflammatory chemokines in response to PIC stimulation. **A)** Relative expression of CCL2 and CXCL8 from HSF stimulated by PIC100μg/mL for 6 hours and 24 hours in the absence and presence of MTX 1μM treatment was assessed by qRT-PCR. **B)** HSF were exposed to PIC 100μg/mL and treated or not with MTX 1μM. Supernatants were harvested after 6 hours and 24 hours and levels of CCL2 and CXCL8 were quantitated by ELISA assay. All experiments were done in quadruplicates and results are expressed as mean ± standard error. *: p-values ≤ 0.05, **: p-values ≤ 0.01, ***: p-values ≤ 0.001 and ****: p-values ≤ 0.0001.

Chemokines play an important role in the pathogenesis of aseptic and septic arthritides through their ability to recruit and activate a wide range of leukocytes [39]. We therefore decided to evaluate the capacity of dsRNA PIC to induce the expression of CCL2 (MCP1) and CXCL8 (IL8) and the potential of MTX to affect proinflammatory chemokines expression. We have first investigated by qRT-PCR the effects of individual and combined treatments of PIC and MTX on mRNA levels of CCL2 and CXCL8 in HSF (**Fig 3**A).

The relative expression of CCL2 was not significantly affected at 6h after PIC exposure. In contrast, we observed a significant increase up to 20 fold of CCL2 mRNA levels at 24h (5.8x 10^+0^ ± 2.74x10^+0^, p<0.01) *versus* (2.92x 10^‐1^ ± 1.54x10^-1^) in control cells. CXCL8 gene expression was significantly increased at 6h and 24h in response to PIC stimulation. More than 40 fold increase was observed at 6h (9.98x 10^‐1^ ± 6.47x10^-1^, p<0.05) *versus* (2.28x 10^‐2^ ± 1.80x10^-3^) and more than 400 fold increase was observed at 24h (9.37x 10^+0^ ± 5.75x10^+0^, p<0.05) *versus* (2.08x 10^‐2^ ± 2.53x10^-2^) in CXCL8 mRNA levels after PIC exposure.

When HSF were treated with MTX alone, no significant difference in CCL2 and CXCL8 mRNA levels was noticed at 6h and 24h. Moreover, MTX did not affect the induction of CCL2 and CXCL8 mRNA levels after PIC treatment.

We next decided to investigate whether PIC and MTX treatment can affect CCL2 and CXCL8 protein secretion from HSF. Cells were exposed to PIC in the presence or not of MTX treatment and the production of proinflammatory chemokines CCL2 and CXCL8 in cell culture supernatants was monitored by ELISA.

When exposed to PIC (Fig 3B), HSF significantly increased CCL2 and CXCL8 release in cell culture supernatants at 6h and 24h. At 6 hours, CCL2 protein levels were (9383pg/mL ± 1893, p<0.001) with a fold change of 7 compared to control (1279 pg/mL ± 563). PIC treatment also increased CXCL8 (456pg/mL ±2 41, p<0.05) with a mean fold change of 5, when compared to control cells (84pg/mL ± 1.6). At 24 hours, we observed a 6 and 32 fold increases in CCL2 and CXCL8 protein levels, respectively following PIC treatment.

After MTX 1μM treatment alone, CCL2 and CXCL8 protein expressions were not affected at 6h and 24h. Moreover, MTX treatment did not modulate the PIC-dependent upregulation of CCL2 and CXCL8 release by HSF.

### MTX does not affect the expression of proinflammatory cytokines, IL-6 and IL1β by HSF

The role of osteoclast formation in arthritis and bone erosion has been well described and it was already reported that HSF played an important role on bone erosion through their ability to secrete a large panel of cytokines such as IL1β, IL-6, M-CSF, GM-CSF and RANKL [21,26,40]. We first tested and validated that the expression of three major osteoclastogenic factors by HSF, RANKL, M-CSF and GM-CSF was upregulated in response to IL1β stimulation (**S1 Fig**) [40–42]. As a mean of controlling for IL1β and PIC stimulatory activities on HSF, we tested and validated that both treatments increased the expression of CD55 by HSF as previously described [43] (**S2 Fig**).

**Fig 4:**
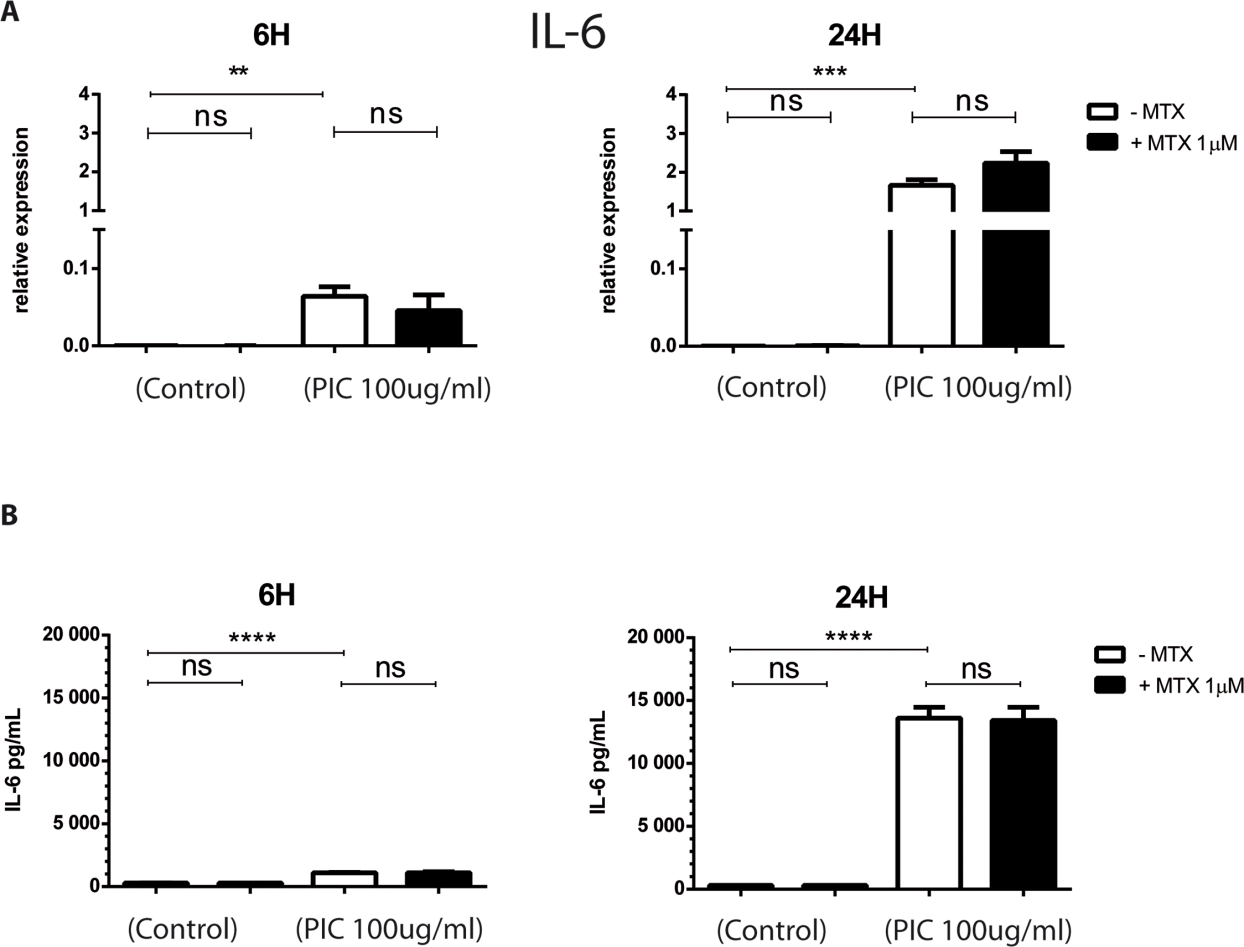
MTX treatment has not effect on the expression of IL-6 cytokine in HSF exposed to PIC. HSF were stimulated by PIC 100μg/mL and treated or not with MTX 1μM. **A)** IL-6 mRNA levels from HSF stimulated by PIC100μg/mL for 6 hours and 24 hours in the absence and presence of MTX 1μM treatment were evaluated by qRT-PCR. **B)** Supernatants were harvested after 6 hours and 24 hours and levels of IL-6 were measured by ELISA assay. All experiments were done in quadruplicates and results are expressed as mean ± standard error. *: p-values ≤ 0.05, **: p-values ≤ 0.01, ***: p-values ≤ 0.001 and ****: p-values ≤ 0.0001.

Exposure to PIC strongly increased IL-6 gene expression in HSF at 6h and 24h (**Fig 4**A). IL-6 mRNA levels were (6.4x 10^−2^ ± 2.16x10^-2^, ≤ 0.01) at 6h and (1.66x 10^+0^ ± 2.55x10^-1^, ≤ 0.001) at 24h when compared to control cells (6h, 2.98x 10^‐4^ ± 2.24x10^-4^); (24h, 4.61x 10^‐4^ ± 7.15x10^-5^). No significant difference in IL-6 mRNA levels was observed after MTX treatment alone or combined with PIC exposure. The upregulation of IL-6 expression after PIC exposure was confirmed at the protein level by ELISA assay at 6h and 24h (Fig 4B): For instance, PIC stimulation significantly increased IL-6 concentration (13609 pg/mL ± 1720, ≤ 0.0001) compared to unstimulated cells (305pg/mL ± 10) with a fold change of 45 at 24h after treatment. MTX treatment had no significant effect on IL-6 release in HSF culture supernatant in control cells as well as after PIC stimulation at 6h and 24h.

IL1β relative expression was also highly upregulated at 24h in response to PIC stimulation (2.27x10^+0^ ± 2.92x10^-1^, p≤ 0.001) *versus* (9.37x10^-4^±1.8x10^-4^) in control cells, corresponding to more than 2400 fold increase (**S3 Fig**).

### MTX does not modulate the expression of GM-CSF in response to PIC stimulation

**Fig 5:**
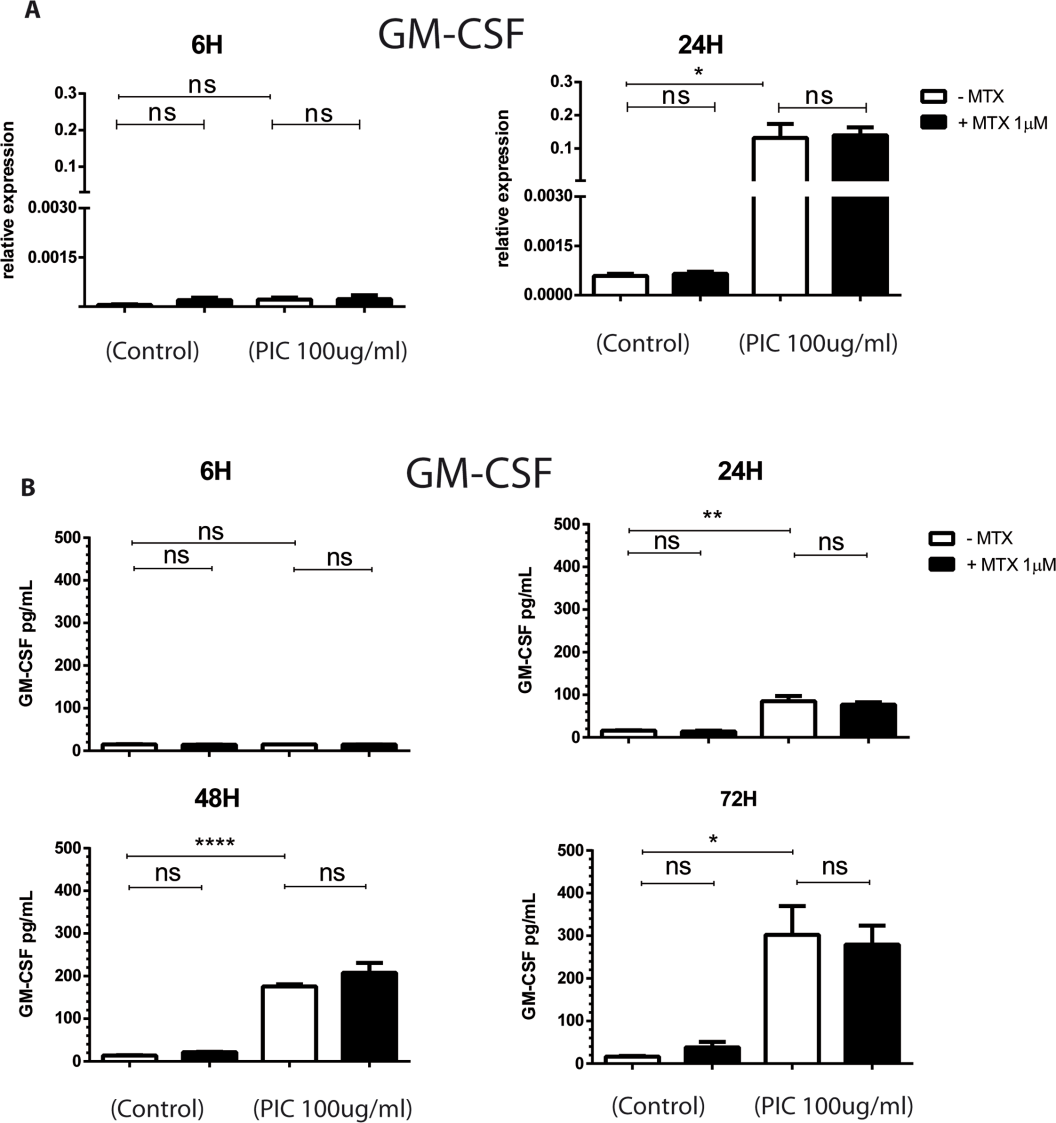
MTX treatment does not affect the expression of GM-CSF in HSF stimulated with PIC. HSF were exposed to PIC 100μg/mL and treated or not with MTX 1μM. **A)** GM-CSF mRNA levels from HSF stimulated by PIC100μg/mL in the absence and presence of MTX 1μM treatment were assessed by qRT-PCR. **B)** Levels of GM-CSF in HSF culture supernatants harvested after 6 hours, 24 hours, 48 hours and 72 hours were measured by ELISA assay. All experiments were done in triplicates and results are expressed as mean ± standard error. *: p-values ≤ 0.05, **: p-values ≤ 0.01, ***: p-values ≤ 0.001 and ****: p-values ≤ 0.0001.

M-CSF and GM-CSF, the main factors of monocytes/macrophages survival, were shown to be involved in synovial inflammation and joint destruction [40,44,45] and higher levels of GM-CSF have been reported to be associated with persistent arthralgia during CHIKV infection [27]. We have evaluated the effects of PIC and MTX treatments on M-CSF and GM-CSF mRNA expression by qRT-PCR and on GM-CSF protein expression by ELISA assay (**Fig 5** and S3 Fig).

PIC exposure did not affect GM-CSF relative mRNA expression at 6h whereas we observed a significant upregulation in GM-CSF and M-CSF mRNA levels at 24h (1.32x10^‐1^ ± 7.33x10^-2,^, p<0.05) and (1.19x 10^+0^ ± 2.49x10^-1,^, p<0.01), respectively as compared to control cells (5.87x10^-4^ ± 1.07x10^-4^) and (6.44x10^-2^ ± 2.18x10^-2^), respectively. MTX did not significantly affect M-CSF and GM-CSF gene expression when used alone or together with PIC.

We next investigated kinetic changes of GM-CSF production in response to PIC stimulation. Protein levels were monitored in HSF culture supernatants from 6h to 72h. We found that GM-CSF production started to increase at 24h (85 pg/mL ± 22, ≤ 0.01) compared to unstimulated cells (16pg/mL ± 1) with a fold change of 5 and reached higher levels at 48h (176pg/mL ± 9, ≤ 0.0001) corresponding to a fold increase of 12 and at 72h (302pg/mL ± 116, ≤ 0.05) with a fold change of 18. When HSF were exposed to MTX 1μM treatment alone, no significant effect was observed on GM-CSF release in cell culture supernatant at 6h, 24h, 48h and 72h. MTX treatment did not affect the upregulation of GM-CSF release after PIC exposure.

### MTX does not affect the expression of RANKL by HSF

**Fig 6:**
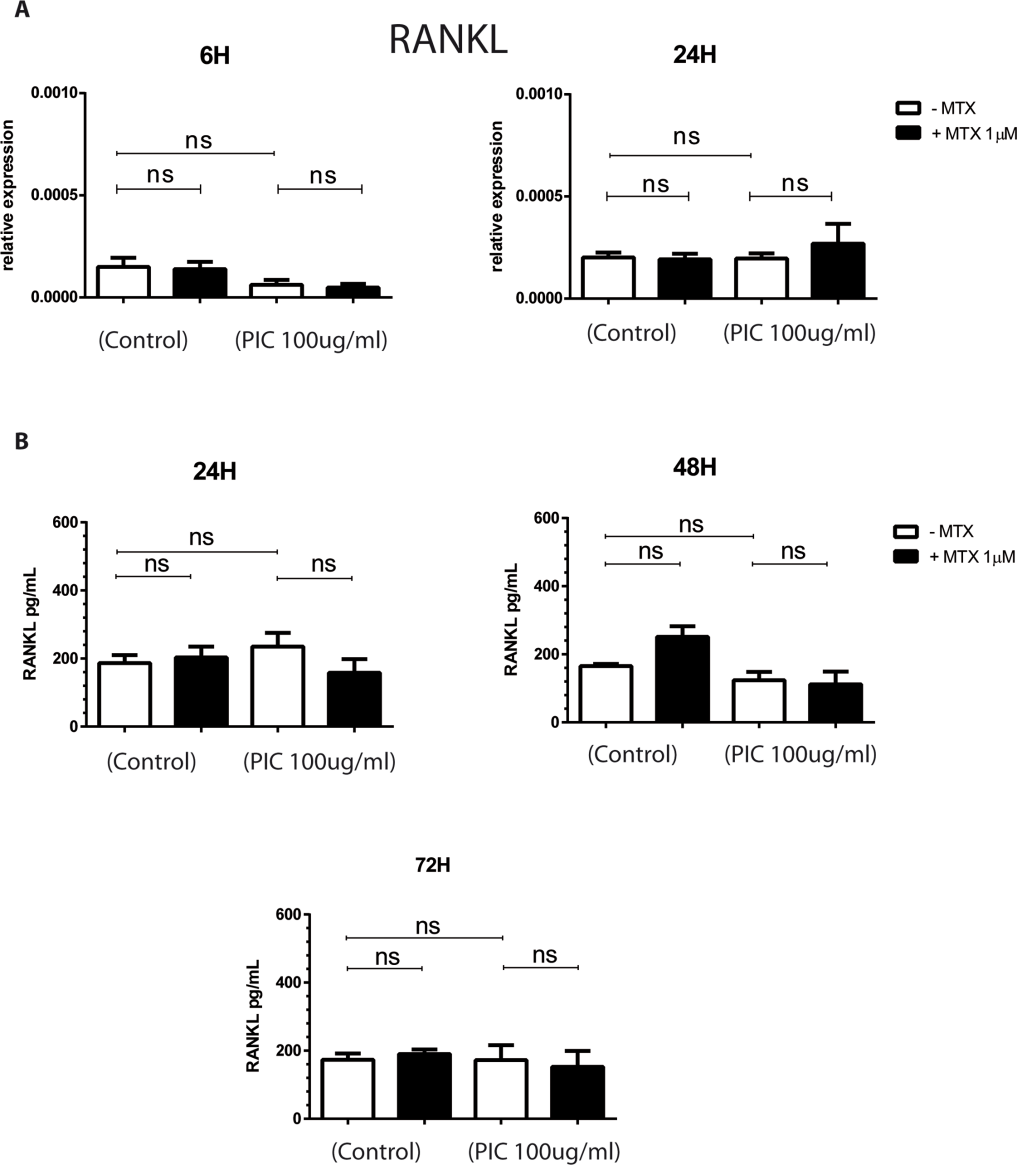
MTX treatment does not modulate the expression of RANKL in HSF exposed to PIC. **A)** mRNA levels of RANKL from HSF stimulated by PIC100μg/mL in the absence and presence of MTX 1μM treatment were measured by qRT-PCR. **B)** Culture supernatants were harvested after 24 hours, 48 hours and 72 hours and RANKL protein levels were measured by ELISA assay. All experiments were done in triplicates and results are expressed as mean ± standard error. *: p-values ≤ 0.05, **: p-values ≤ 0.01, ***: p-values ≤ 0.001 and ****: p-values ≤ 0.0001.

RANKL has been identified as a crucial regulator and promoter of osteoclastogenesis and bone erosion. RANKL is made as a membrane-bound molecule that can be released from the cell surface after proteolytic cleavage by ADAM17 [46,47]. We investigated the ability of PIC and MTX to affect RANKL and ADAM17 expression by HSF. RNA levels of RANKL were assessed by qRT-PCR at 6h and 24h after PIC exposure in presence or absence of MTX 1μM treatment. RANKL release in cell culture supernatant was measured by ELISA assay (**Fig 6**).

Unexpectedly, PIC was not able to induce RANKL mRNA expression in HSF at 6h and 24h. In contrast, ADAM17 gene expression was significantly upregulated by PIC at 24h (1.61x10^+0^ ± 4.39x10^-1,^, p<0.05) with a fold change of 3 compared to control cells (4.97x10^‐1^ ± 2.03x10^-1^) (S3 Fig). MTX 1μM treatment alone or combined with PIC did not affect RANKL mRNA levels.

We next evaluated RANKL secretion in HSF culture supernatants at 24h, 48h and 72h after PIC 100μg/mL exposure in the presence or not of MTX 1μM. PIC had no effect on RANKL protein levels and MTX did not affect significantly RANKL release in all tested conditions, alone or together with PIC.

### MTX treatment does not affect CHIKV replication in HSF

**Fig 7:**
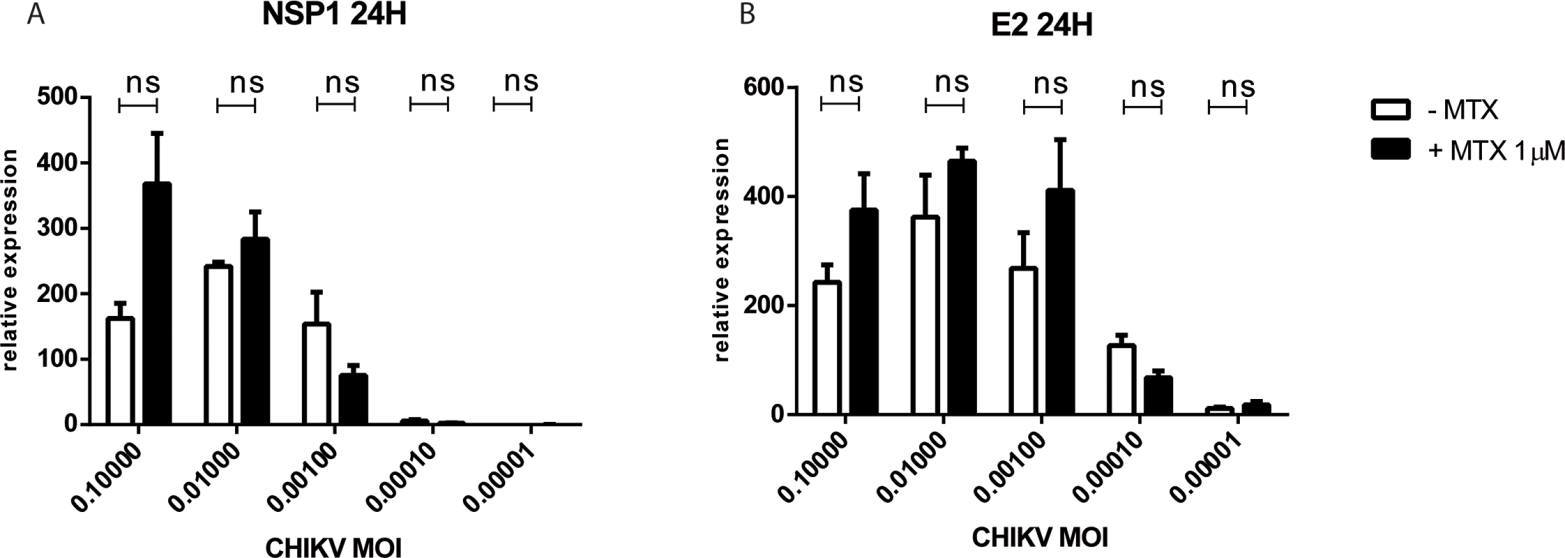
MTX treatment does not affect CHIKV replication in HSF. Relative expression of viral NSP1 (A) and E2 (B) mRNA from HSF infected with different MOIs of CHIKV for 24 hours after exposure or not to MTX 1μM treatment (qRT-PCR data). Experiments were done in triplicates and results are expressed as mean ± standard error. *: p-values ≤ 0.05, **: p-values ≤ 0.01, ***: p-values ≤ 0.001 and ****: p-values ≤ 0.0001.

We next questioned whether MTX treatment could directly affect viral replication in HSF infected by CHIKV following exposure at different MOIs. As shown in **Fig 7**, no significant difference in NSP1 and E2 RNA levels was noted after MTX 1μM treatment of HSF exposed to CHIKV.

### MTX treatment does not modulate the antiviral innate immune response mobilized by CHIKV infection

To study the effect of MTX treatment on the antiviral innate immune responses of HSF exposed to CHIKV, we evaluated by qRT-PCR the expression of IFN β and ISG15 antiviral genes at 24H post-infection.

**Fig 8:**
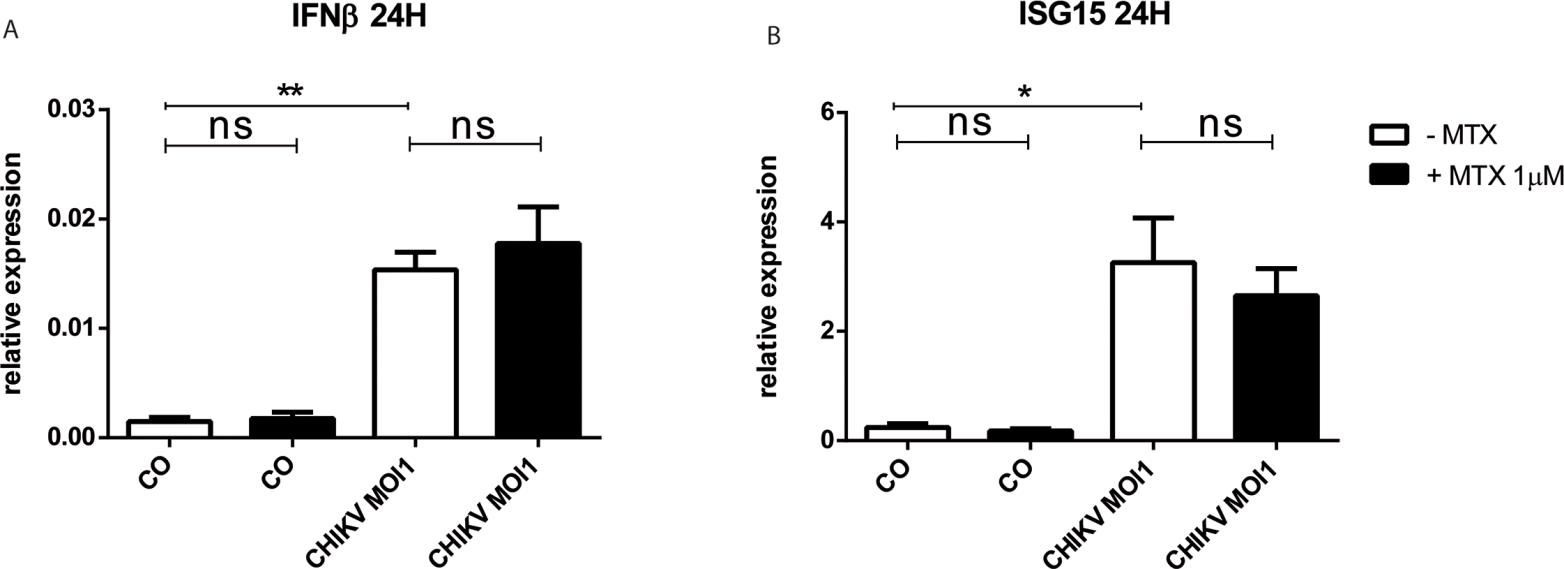
MTX treatment does not affect the expression of IFN β and ISG15 antiviral innate immune genes in response to CHIKV infection. Relative expression IFN β (A) and ISG15 (B) mRNA from HSF infected with CHIKV at MOI1 for 24 hours after exposure or not to MTX 1μM as assessed by qRT-PCR. Experiments were done in triplicates and results are expressed as mean ± standard error. *: p-values ≤ 0.05, **: p-values ≤ 0.01, ***: p-values ≤ 0.001 and ****: p-values ≤ 0.0001.

In response to CHIKV MOI of 1, the relative expression of IFN β was significantly increased in HSF at 24H post-infection (1.54x10^‐2^ ± 2.75x10^-3^, p<0.01) with a fold change of 10 compared to mock-infected control cells (1.48x10^‐3^ ± 6.69x10^-4^) (**Fig 8**). Moreover, CHIKV infection significantly upregulated the expression of ISG15 mRNA at 24H post-infection with a fold change of 14. MTX 1μM did not affect significantly the relative expression of IFN β and ISG15 in mock-infected control cells as well as after CHIKV MOI1 infection.

## Discussion

We argued that PIC treatment could be used essentially to mimic the effect of viral dsRNA present in the joint during the chronic phase of CHIKV for the following reasons [11]. First, and of critical note, the presence of viral dsRNA in the joint during the chronic phase of CHIKV was revealed in a human clinical case report 18 months post-infection [11]. A similar observation was reported in a macaque model of CHIKV infection, with the persistence of CHIKV RNA up to 44 days post-infection [10]. Using a murine model of a long lasting CHIK infection established by Hawman et al., the authors detected the release of dsRNA up to 112 days post-infection [48]. In addition, it is now well established that PIC can induce arthritis in 3 days post-treated mice [35] and that dsRNA has been detected in the synovial fluid and serum of patients suffering from RA [42]. While the source of dsRNA was not identified by Bokareva et al., it was established that patients with erosive disease had significantly higher levels of dsRNA in synovial fluid than patients diagnosed with non-erosive RA.

MTX has been initially identified as an anti-metabolite drug. Several reports have described the effects of MTX on the inhibition of synovial fibroblast invasion and proliferation [49,50]. To examine whether MTX may induce HSF cell death we used one cytotoxic assay. We demonstrated that MTX treatment at the concentration of 1μM (therapeutic dose of RA as well as CHIKV-induced chronic arthritis) or even at 10μM did not cause HSF necrosis.

We next studied the effect of PIC on the antiviral response of HSF. First, we observed that HSF constitutively expressed dsRNA receptors, ie. MDA5 and RIG-I. In agreement with our findings, previous studies have shown that FLS expressed MDA5, and RIG-I [43,51]. PIC is an agonist particularly of MDA5 and has been shown to stimulate synovial cells to induce interferon type I production [43,52]. We found that the transcription levels of MDA5 and RIG-I were upregulated in response to PIC stimulation. Moreover, PIC induced the expression of high levels of IFNβ. We next questioned whether MTX treatment may affect the aforementioned antiviral immune response. We found that MTX increased MDA5 mRNA levels. However, MTX treatment had no effect on RIG-I, IFNβ and ISG15 mRNA levels.

CCL2 and CXCL8 are major chemokines involved in modulating the recruitment of immune cells in the inflamed joint [39]. We demonstrated that HSF constitutively express CCL2 and CXCL8 albeit at low levels. These results are in line with earlier studies showing that synovial fibroblasts display low constitutive expression of CCL2 and CXCL8 [53,54]. We observed that PIC significantly upregulated CCL2 and CXCL8 mRNA levels and protein secretion by HSF. Moon et al. showed that PIC stimulation increased CXCL8 expression at both the mRNA and protein levels in RA synovial fibroblasts [55]. Interestingly, CCL2 and CXCL8 were detected in the synovial tissue of RA patients and CXCL8 expression correlated with the development of clinical signs and synovial inflammation [56]. We herein demonstrated that MTX at 1μM did not affect CCL2 and CXCL8 expression in HSF in basal conditions as well as after PIC exposure and hence may not promote inflammatory functions of synovial cells.

A plethora of pro-inflammatory cytokines such as IL1β, IL-6 and M-CSF were documented to promote osteoclast differentiation and bone resorption [21]. They may act by increasing production of RANKL, identified as key mediator of osteoclastogenesis, or by inducing the development of osteoclast precursors. Here, we demonstrated that HSF express low mRNA and protein levels of IL-6 in basal conditions and that IL-6 expression was strongly enhanced in response to PIC stimulation. MTX 1μM had no inhibitory effect on IL-6 mRNA expression and protein secretion by HSF stimulated or not with PIC. We also demonstrated that GM-CSF was expressed constitutively at low levels by HSF and its expression was significantly upregulated by PIC. These results are in line with previous studies showing that synovial fibroblasts expressed weakly GM-CSF at basal conditions [40]. The involvement of GM-CSF in joint inflammation and destruction has been demonstrated in animal model of RA [44]. We found that MTX 1μM did not modulate constitutive and PIC-induced GM-CSF expression by HSF. It has been reported that MTX can inhibit GM-CSF production in whole blood culture from RA patients [57]. When applied on human synovial sarcoma cell line, used as an *in vitro* model of RA, MTX significantly decreased GM-CSF secretion in culture supernatants. However, these results are difficult to compare to ours because MTX was used at much higher concentrations of 0.1 and 1mg/mL corresponding respectively to 220 μM and 2200 μM [58].

RANKL is the central mediator of osteoclast development. It is considered as an essential factor for osteoclast activation and survival [23]. Here we demonstrated that our HSF constitutively express RANKL mRNA and protein at low levels. A similar observation was found by Tunyogi-Csapo and colleagues. They demonstrated that HSF express RANKL mRNA and are sources of RANKL production [59]. They also reported that RA HSF may significantly contribute to bone resorption through the modulation of RANKL production in inflamed joints. An interesting finding of our study was the observation that PIC alone was not able to increase RANKL mRNA and protein expression in cultured HSF. IL-6 and IL1β were reported to induce RANKL expression in synovial fibroblasts [41]. Although we found that PIC upregulated IL1β and IL-6 expression, these events did not translate into an increased RANKL expression by HSF. Of note, Kim et al. have found that PIC significantly upregulated RANKL mRNA levels in RA HSF but not in OA HSF and normal skin fibroblasts [60]. We have been using primary HSF and it will be interesting to address the fine mechanisms by which PIC can nevertheless control RANKL expression and which may involve specific signaling pathways present in inflamed RA fibroblasts but not in naïve conditions. We found that MTX at the concentration of 1μM did not modulate RANKL expression in HSF and, hence, may not affect the bone tissue repair mechanisms.

We also analyzed the capacity of MTX to modulate CHIKV infection and replication in HSF. We tested CHIKV at different MOI and particularly at very low MOI in order to mimic in situ tissue settings of patients chronically infected. Our data showed that MTX did not affect CHIKV replication. These *in vitro* data are in agreement with results obtained in mice where it was shown that the CHIKV load was not increased in target tissues when mice were treated with the immunosuppressive drug MTX (0.3 mg/kg, intraperitoneally) [61]. In contrast, this situation might be different for another alphavirus as shown in one study by Taylor et al [62]. It was shown that MTX caused a rapid development of severe disease in treated mice with a significant increase in viral titer in sera and quadriceps. It is possible that MTX may have an effect on the infectious process during the acute phase of alphaviral infection. Our paradigm has been to address the role of MTX in chronic settings at the distance from the initial infection and to better address the therapeutic window of MTX which is clearly in chronic but not acute phases of chikungunya.

In conclusion, we consider that we have been able to model the context of CHIKV persistence in the joint of patients suffering from chronic injuries using the PIC molecule. Moreover, we have been addressing for the first time the role of the immunosuppressive drug MTX in the overall antiviral, inflammatory and pro-osteoclastogenic responses which may all be in action in the joint of patients suffering from CIR. Scientists and clinicians have been concerned that the immunosuppressive drug could contribute to the resurgence of the virus in patients. Critically, our study revealed for the first time that MTX treatment is likely to be safe and did not affect the antiviral immune and inflammatory responses of HSF. MTX had no modulatory effect on the expression of several pro-osteoclastic cytokines by HSF and which are involved in bone tissue repair.

Further studies are warranted to address whether MTX could affect the expression of inflammatory and co-stimulatory molecules involved in the recruitment and activation of T cells and monocytes and which are present in the synovial tissue of patients with CIR post-CHIK.

**Fig 9:**
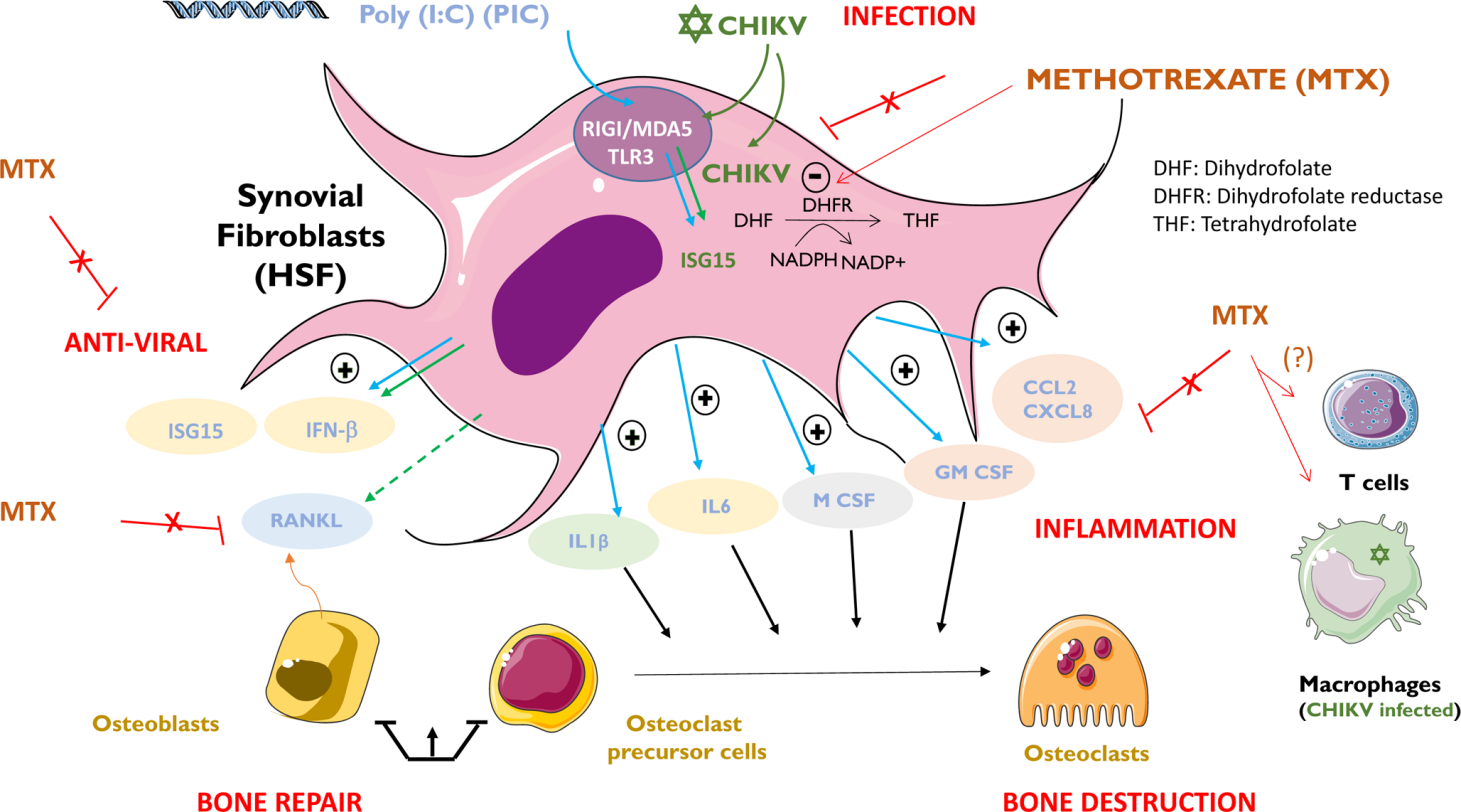
Immunosuppressive MTX drug at micromolar concentrations does not affect the antiviral, inflammatory and pro-osteoclastogenic functions of HSF. Chikungunya virus (CHIKV, ✡) is a single strand RNA virus capable of replicating at high levels in human synovial fibroblasts (HSF) and to contribute to chronic arthritis through ill-characterized mechanisms. CHIKV will be recognized by RIG-I and MDA5 pattern recognition receptors to initiate the canonical interferon-type I dependent antiviral response and to control infection. Poly I:C (PIC) is a viral RNA mimetic. Upon stimulation by CHIKV (or PIC), HSF will express antiviral genes (e.g. ISG15) as well as a myriad of proinflammatory molecules (interleukins/IL1, chemokines/CCL2) and growth factors involved in bone repair (e.g. M-CSF, GM-CSF). CHIKV infection (but not PIC stimulation) also is known to upregulate the expression of RANKL involved in osteoclastogenic activities and bone destruction. We herein have shown that methotrexate (MTX) a well-known therapeutic agent (DHFR inhibitor) against arthritis does not interfere with the antiviral and bone repair mechanisms essential to regulate CHIKV-induced chronic joint diseases. Critically, MTX does not aggravate the level of CHIKV replication in HSF. Macrophages (latently infected by CHIKV) and recruited CD4 T lymphocytes will contribute to chronic arthritis post-CHIKV but a plausible role of MTX on these cells remains to be addressed.

All of our findings are summarized and illustrated in **Fig 9**.

## Supporting information

S1 Fig GM-CSF, M-CSF, RANKL and ADAM17 expression in HSF in response to LPS and proinflammatory cytokines stimulation. Relative expression of GM-CSF, M-CSF, RANKL and ADMA17 mRNAs from HSF stimulated for 24 hours was assessed by qRT-PCR. HSF were non stimulated (Control) or stimulated with LPS 1μg/mL, IL1β 50ng/mL and TNFα 50ng/mL. n = 3. Results are expressed as mean ± standard error. *: p-values ≤ 0.05, **: p-values ≤ 0.01.
(TIF)

S2 Fig Characterization of human synovial fibroblast (cluster of differentiation, CD) markers. FACS analysis to evaluate the expression levels of HSF cell markers. The data are expressed as mean fluorescence intensities (MFI). The grey box represents the level of background staining obtained with isotype negative control antibodies. Cells were non stimulated (Control) or were treated with PIC 100μg/mL and IL1β 50ng/mL for 24 hours. n = 3. Results are expressed as mean ± standard error. *: p-values ≤ 0.05.
(TIF)

S3 Fig MTX does not modulate the expression of IL1β, M-CSF and ADAM17 in HSF exposed to PIC. To analyze the expression profile of HSF IL1β and M-CSF pro-osteoclastogenic cytokine genes and ADAM17 gene, cells were stimulated with PIC 100μg/mL in the presence or not of MTX 1μM for 24 hours. Relative expression was analyzed by qRT-PCR. n = 3. Results are expressed as mean ± standard error. *: p-values ≤ 0.05, **: p-values ≤ 0.01, ***: p-values ≤ 0.001.
(TIF)
