# Supplementary figures and images for: Immunomodulatory drug methotrexate used to treat patients with chronic inflammatory rheumatisms post-chikungunya does not impair the synovial antiviral and bone repair responses
Source: PLoS Negl Trop Dis. 2018 Aug 3;12(8):e0006634. doi: 10.1371/journal.pntd.0006634 (PMC6093699; doi:10.1371/journal.pntd.0006634)

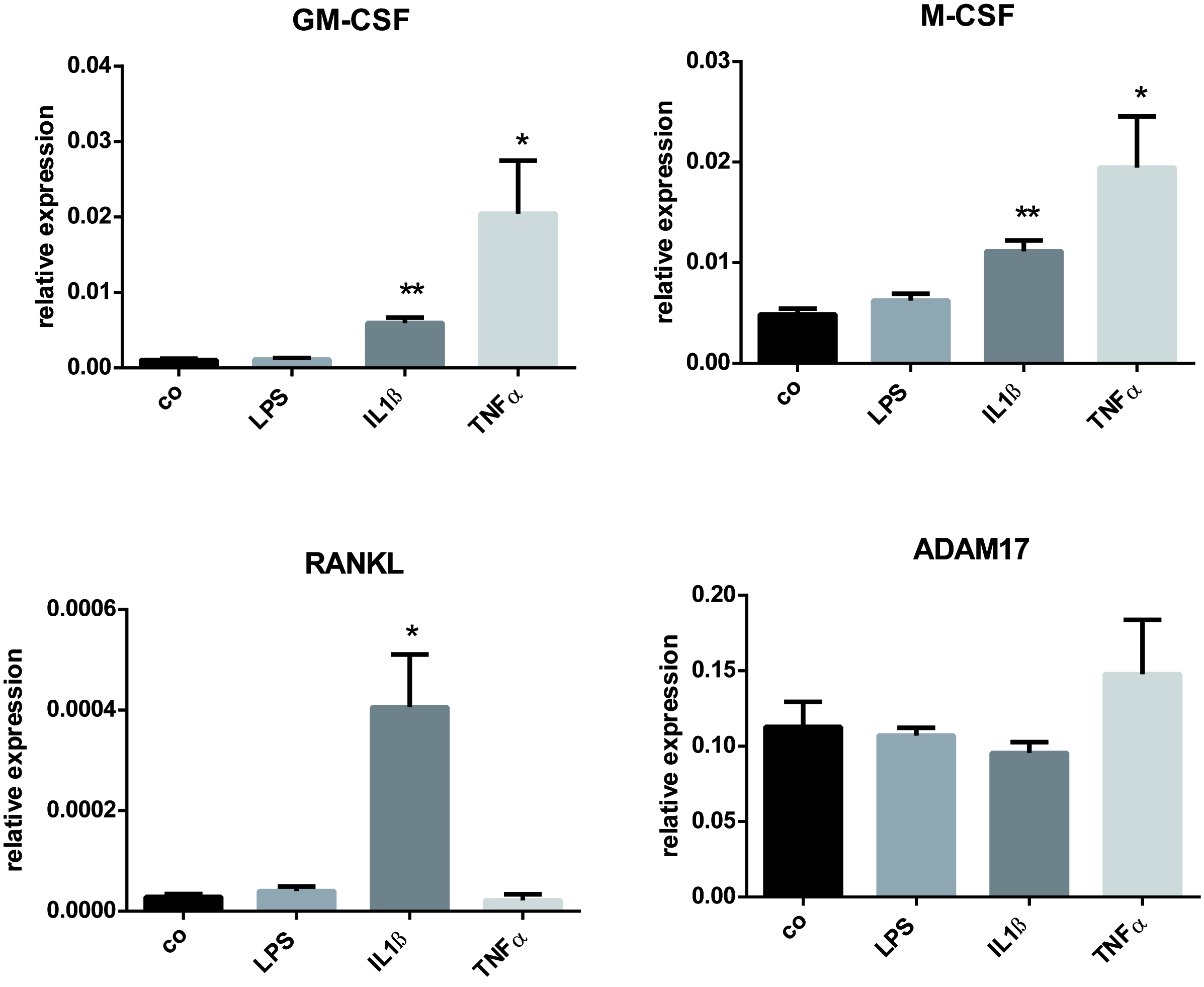

Supplement: S1 Fig — Relative expression of GM-CSF, M-CSF, RANKL and ADMA17 mRNAs from HSF stimulated for 24 hours was assessed by qRT-PCR. HSF were non stimulated (Control) or stimulated with LPS 1μg/mL, IL1β 50ng/mL and TNFα 50ng/mL. n = 3. Results are expressed as mean ± standard error. *: p-values ≤ 0.05, **: p-values ≤ 0.01. (TIF) [file pntd.0006634.s001.tif]

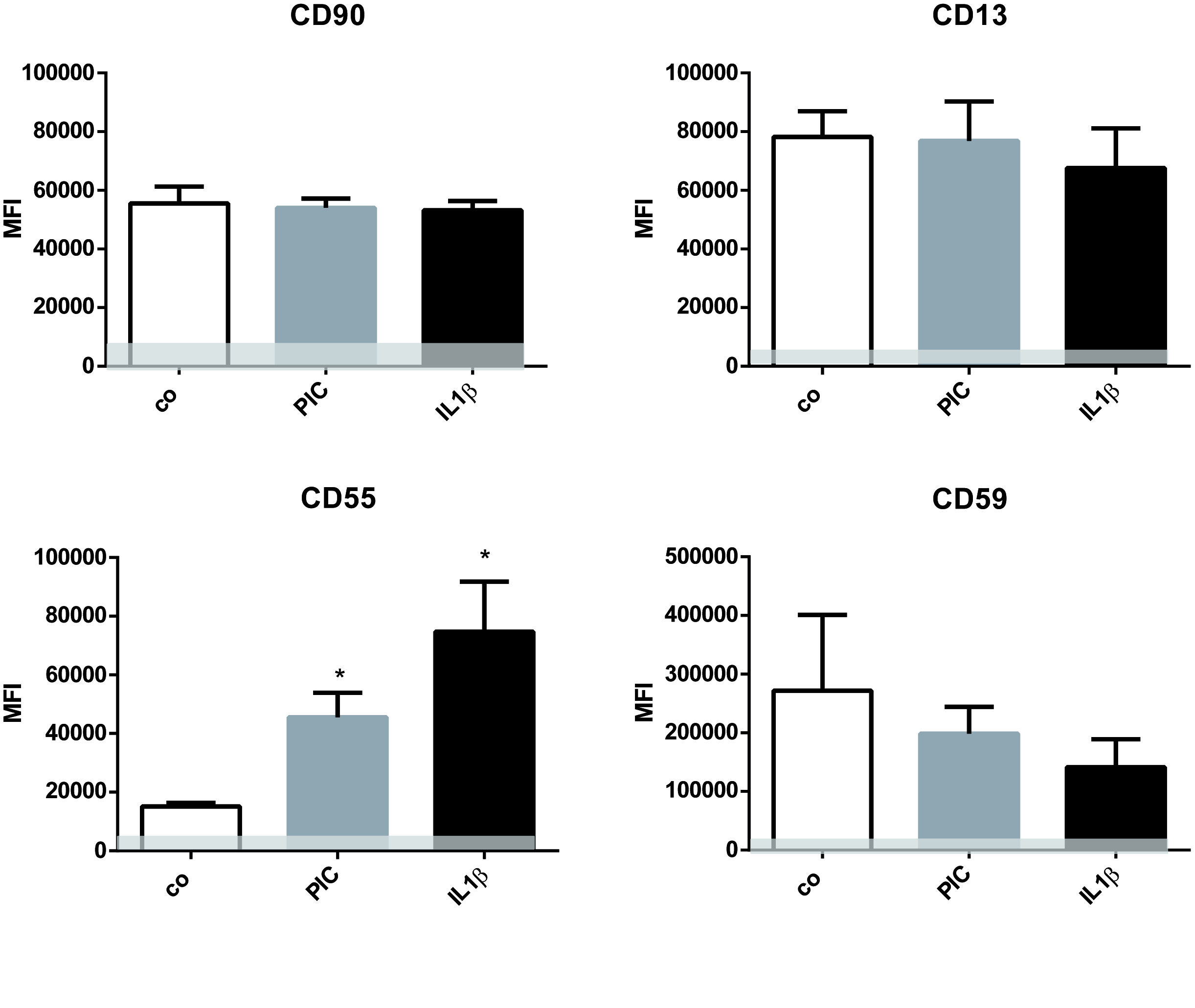

Supplement: S2 Fig — FACS analysis to evaluate the expression levels of HSF cell markers. The data are expressed as mean fluorescence intensities (MFI). The grey box represents the level of background staining obtained with isotype negative control antibodies. Cells were non stimulated (Control) or were treated with PIC 100μg/mL and IL1β 50ng/mL for 24 hours. n = 3. Results are expressed as mean ± standard error. *: p-values ≤ 0.05. (TIF) [file pntd.0006634.s002.tif]

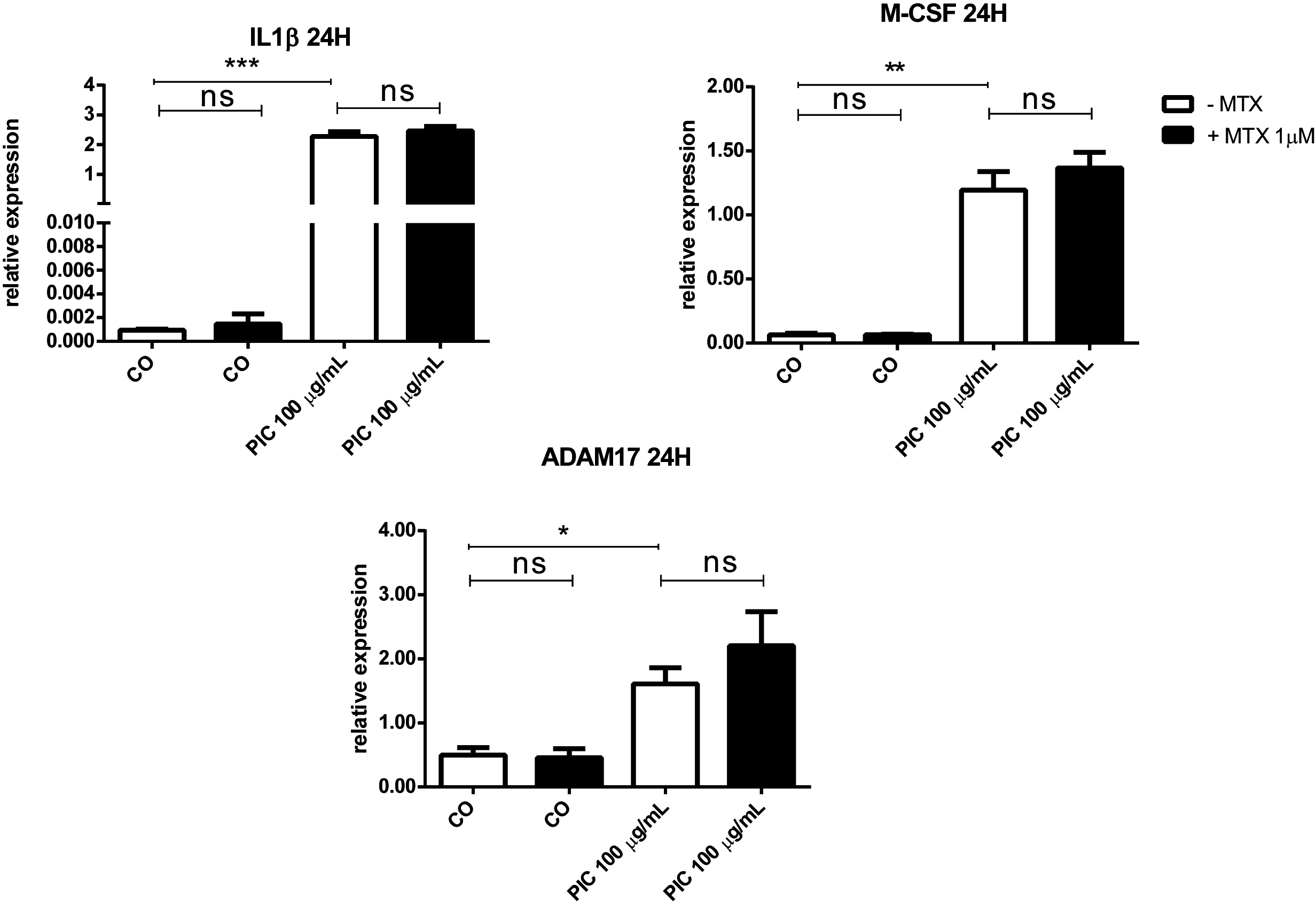

Supplement: S3 Fig — To analyze the expression profile of HSF IL1β and M-CSF pro-osteoclastogenic cytokine genes and ADAM17 gene, cells were stimulated with PIC 100μg/mL in the presence or not of MTX 1μM for 24 hours. Relative expression was analyzed by qRT-PCR. n = 3. Results are expressed as mean ± standard error. *: p-values ≤ 0.05, **: p-values ≤ 0.01, ***: p-values ≤ 0.001. (TIF) [file pntd.0006634.s003.tif]
